# Identification of novel AKT1 inhibitors from *Sapria himalayana* bioactive compounds using structure-based virtual screening and molecular dynamics simulations

**DOI:** 10.1186/s12906-024-04415-3

**Published:** 2024-03-07

**Authors:** Laldinfeli Ralte, Hmingremhlua Sailo, Rakesh Kumar, Laldinliana Khiangte, Nachimuthu Senthil Kumar, Yengkhom Tunginba Singh

**Affiliations:** 1 Department of Botany, Mizoram University, Aizawl, Mizoram 796004 India; 2 Department of Biotechnology, Mizoram University, Aizawl, Mizoram 796004 India; 3 Present Address: Department of Life Sciences (Botany), Manipur University, Imphal, Manipur 795003 India

**Keywords:** *Sapria himalayana*, Cytotoxicity, Network pharmacology, Molecular docking, Molecular dynamic simulation

## Abstract

Through the experimental and computational analyses, the present study sought to elucidate the chemical composition and anticancer potential of *Sapria himalayana* plant extract (SHPE). An in vitro analysis of the plant extract was carried out to determine the anticancer potential. Further, network pharmacology, molecular docking, and molecular dynamic simulation were employed to evaluate the potential phytochemical compounds for cervical cancer (CC) drug formulations. The SHPE exhibited anti-cancerous potential through inhibition properties against cancer cell lines. The LC-MS profiling showed the presence of 14 compounds in SHPE. Using network pharmacology analysis, AKT1 (AKT serine/threonine kinase 1) is identified as the possible potential target, and EGFR (Epidermal Growth Factor Receptor) is identified as the possible key signal pathway. The major targets were determined to be AKT1, EGFR by topological analysis and molecular docking. An in silico interaction of phytoconstituents employing molecular docking demonstrated a high binding inclination of ergoloid mesylate and Ergosta-5,7,9(11),22-tetraen-3-ol, (3.beta.,22E)- with binding affinities of -15.5 kcal/mol, and -11.3 kcal/mol respectively. Further, MD simulation and PCA analyses showed that the phytochemicals possessed significant binding efficacy with CC protein. These results point the way for more investigation into SHPE compound’s potential as CC treatment.

**Graphical Abstract:**

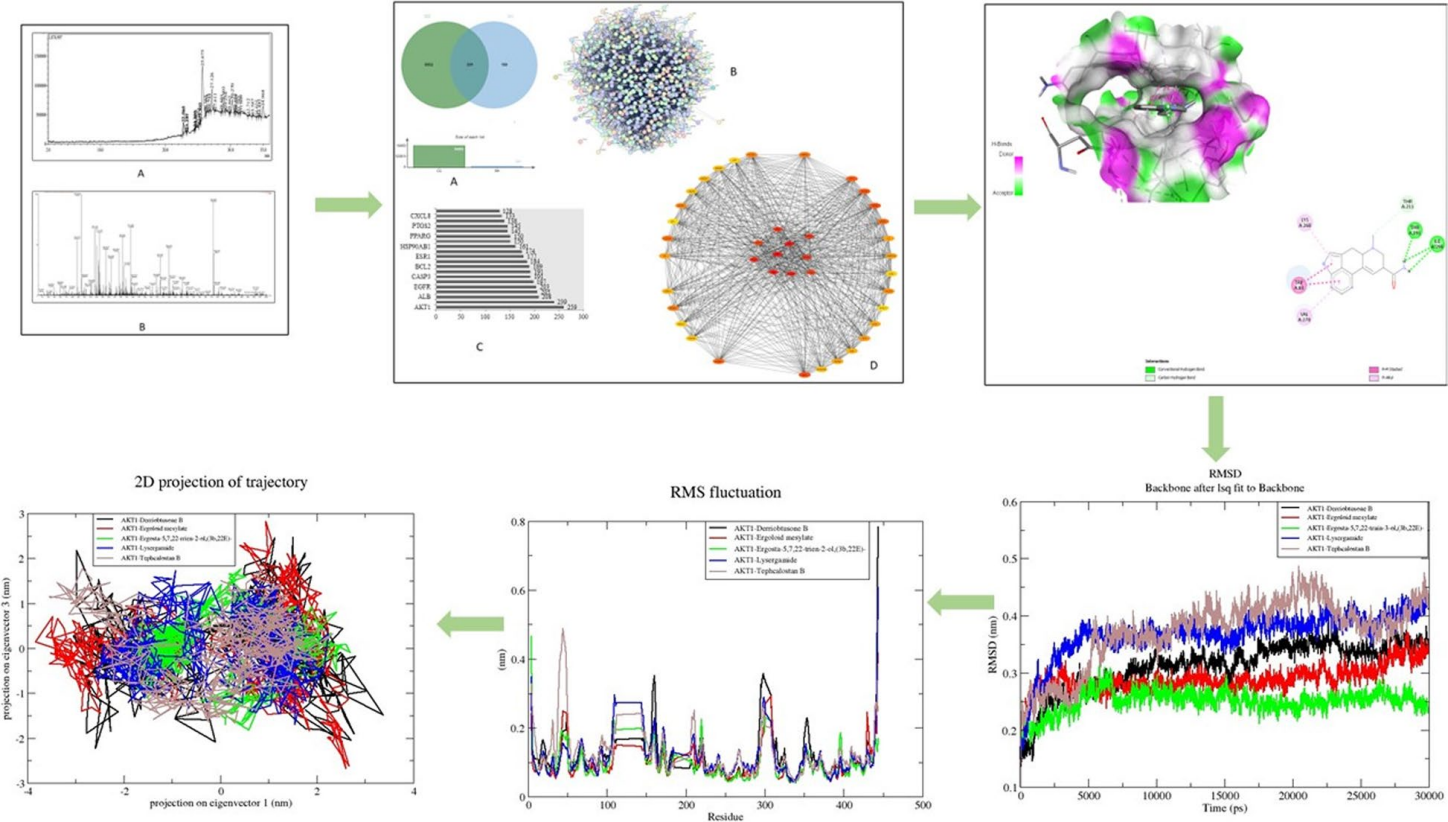

**Supplementary Information:**

The online version contains supplementary material available at 10.1186/s12906-024-04415-3.

## Introduction

The largest cause of morbidity and cancer deaths worldwide is cervical cancer (CC), which is rated fourth in both incidence and mortality [1]. CC accounted for 6.6% and 7.5% of female tumor morbidity and mortality in 2018, with 8,400,000 new cases and 4,200,000 deaths. The greatest rate of cancer in India is found in the north-east (NE) area, which is also plagued by a higher prevalence of cancer risk factors and insufficient cancer treatment options and Mizoram has the second highest incidence of CC in India [2]. When detected, CC is one of the cancers that can be cured the most successfully [3]. Currently, radiation, chemotherapy, targeted therapy, and immunotherapy are the main therapeutic options. However, medication resistance will develop as a result of these treatments and will also struggle with a low response rate [4]. As a result, it is critical to identify a natural extract with low side effects and understand its molecular processes.

Researchers have actively researched drug development utilizing natural resources, and the use of plant-derived compounds is common in cancer research [5]. In contrast to normal cells, natural products have shown selective effects against cancer cells, but their chemical structures can also be used as models to create new medications. These approaches create medications that provide similar or better advantages than existing drugs while having fewer side effects and resistance [6]. *Sapria himalayana* is an endoparasitic plant previously reported from northeastern parts of India [7–11], Myanmar [12], and Thailand [13]. An ethnobotanical study conducted by Wangchuk et al. [14] revealed that the plant was reported by the locals to cure liver diseases and fever [15]. The development of cervical cancer is mostly dependent on the interaction of tumor suppressor proteins p53 and pRB (retinoblastoma) with viral oncoproteins E6 and E7. Dysregulation in cellular adhesion, cellular control, host cell immunomodulation, and genotoxicity is connected with the severity of the disease [16]. Chemotherapeutic drugs, which are not specific to their targets, or more intrusive and costly surgical and ablative methods can be used to treat the disease [17]. Furthermore, millions of patients, especially in underdeveloped nations, have limited access to them. Therefore, the availability of extremely efficient natural therapies targeted against the disease is one of the primary possibilities to treat CC. Hence, the present medicinal plant, which has been traditionally used for the treatment of various ailments was investigated for the biochemical compositions. In the past, a large number of natural and plant-based substances have been found to be promising sources for the creation of cytotoxic agents in the fight against and prevention of cancer [18]. It has also been demonstrated that the active component of *B. aristata*, stigmasterol, has anti-angiogenic and anti-cancer properties via downregulation of VEGFR-2 and TNF-α [19]. Additionally, various compounds such as β-Sitosterol, Lupeol, 3-o-b-galactopyranoside, Quercetin, and Berberine have been found to be the potential inhibitors of CC [20].

The network of regulation of drug-component diseases is built using network pharmacology (NP), which is based on the similarity between drugs in terms of structure and efficacy. This network also takes into account the interaction between biological effector molecules and target molecules in the body as well as joint analysis of genes associated with disease [21]. NP places a strong emphasis on multichannel modulation of signal pathways, which enhances the therapeutic efficacy of medications and lowers their toxic and side effects [21]. As a result, clinical trials for novel drugs are more likely to be successful, which lowers the cost of drug research and development.

This study examined the main active chemical components of SH using NP, investigated the cell viability against cancer cell lines, and its targeted regulation genes, and screened out the optimal target gene sites for the treatment of CC, to clarify the theoretical underpinnings and potential molecular mechanism of SH. Further, we also examined the potential signaling pathways for these genes, as well as the molecular docking, MD simulation, and visualization of the phytochemicals of SH.

## Methodology

### Plant collection

*S. himalayana* was collected from Rullam, Serchhip District, Mizoram, India, and brought to the Department of Botany, Mizoram University. The collected plant was identified morphologically by Dr. Kh. Sadhyarani Devi, Taxonomist, Department of Botany, Mizoram University, and also by NCBI BLAST of the internal transcribed spacer 2 (ITS2) gene sequence (GenBank Accession No. MW788913), and a voucher specimen (MZU/BOT/426) was deposited in the Herbarium of Department of Botany, Mizoram University. All the studied protocols had been ethically evaluated and authorized by the Institution Human Ethical Committee (IHEC) of Mizoram University.

### Sample preparation

The plant material (bud) was air-dried at room temperature till it became completely dry and processed into powdered form. Fifty grams of powder was extracted with 500 ml of methanol using the Soxhlet apparatus for 35 cycles. The extract was concentrated using a water bath at 35°C and the crude extract was stored at 4°C for further use.

### In vitro cytotoxicity assay

#### Cell lines and culture

The cancer cell lines– HeLa (Human Cervical cancer), MCF-7 (Breast Cancer), and K562 (Human Erythroleukemic) cells were obtained from National Centre for Cell Sciences (NCCS), Pune, India, and screened against the extract. The cancer cell lines were cultured in DMEM supplemented with 10% inactivated Fetal Bovine Serum (FBS), penicillin (100 µg/ml), streptomycin (100 µg/ml), and amphotericin B (5 µg/ml) in a humidified atmosphere of 5 % CO_2_ at 37°C until confluent. A trypsin solution (0.2% trypsin, 0.02% EDTA, 0.05% glucose in PBS) was used to dissociate the cells. All the experiments were carried out in triplicates at 96 microtiter plates, using stock culture cultivated in 25 cm^2^ culture flasks.

#### MTT assay

The MTT assay was used to investigate the cytotoxicity of the extract against HeLa, MCF-7, and K562 cell lines. The cell line was grown on a 96-well plate with a cell density 10 x 10^-4^ cells per well in 100 µl of media and incubated for 24 hrs at 37°C in a 5% CO_2_ incubator chamber. The plates were treated with 5% extract (1-200 µg/ml). The untreated cells served as a positive control, whereas cells incubated with 5% methanol served as a blank. Cells in the control group were cultured in a medium containing 0.1% DMSO. All the experiment was carried out in triplicates. After 24 hrs of incubation, the culture media was changed with 20 µl of MTT in each well and incubated for another 4 hrs and the absorbance was taken at 570 nm after the addition of DMSO and each treatment was recorded in triplicate and compared to the untreated cells. Then, the cell viability (%) was calculated as:

$$Cell \,viability\ (\mathrm{\%}) = (Absorbance\, sample-Absorbance \,blank)/ Absorbance \,control-Absorbance\, blank)\times 100$$

The percentage growth of inhibition was also calculated using

$$\mathrm{\% }of\ cell\, inhibition = 100-[(absorbance \,of \,sample-absorbance\, of\, blank)/(absorbance \,of \,control-absorbance\, of \,blank)] \times 100$$

To assess the effects of plant extract, the IC_50_ value was employed, which was the drug dose that reduced the absorbance of treated cells by 50% when compared to untreated cells.

#### Lactate dehydrogenase (LDH) assay

The release of cytoplasmic enzyme, lactate dehydrogenase is caused by cell membrane rupture. One of the symptoms of cellular death is damage to the plasma membrane. To test for this damage, the LDH cytotoxicity assay kit (Thermo Fisher Scientific Inc., Waltham, MA, USA) was used following the manufacturer’s instructions. Briefly stated, the cells were plated in a 96-well plate overnight, subjected to the cytotoxic extracts, and then incubated for 48 hrs. After 180 minutes of incubation at 37°C, 10µl of lysis buffer was added to each well. The reaction mixture was then combined with 50 µl of the supernatant from each well, and the mixture was left in the dark to incubate for 30 minutes at room temperature. Sodium pyruvate was used as a standard. Then, the absorbance was measured at 420 nm and the LDH activity was determined by using the formula:

$$\mathrm{\%\ }Cytotoxicity = Absorbance \,at\, 420 \,nm\, of\, plant\, extract\, treated \,sample/Absorbance\, at\, 420\, nm \,control\times 100-100$$

A triplicate of each extract was tested. Geometric mean IC_50_ values and 95% confidence intervals for the replicates’ geometric means were calculated using log transformed IC_50_ values.

#### Evaluation and screening of phytochemical compounds

GC-MS and LC-MS analyses were performed to evaluate the phytochemical compounds present in SH. SwissADME (www.swissadme.ch), a free web application was used for the in silico ADME screening and drug-likeness evaluation [22].

#### Screening of potential targets

The 3D structure of the compounds was retrieved from the PubChem database (http://pubchem.ncbi.nlm.nih.gov/.) and its target genes were obtained from the Swiss target prediction database (http://www.swisstargetprediction.ch/). The CC-related target genes were obtained from GeneCards (http://GeneCards.org/). Jvenn (http://jvenn.toulouse.inra.fr/) was used to identify the shared targets between the compounds and CC.

#### PPI network construction, GO, and KEGG pathway enrichment analysis

To examine the functional connection between proteins, a protein-protein interaction (PPI) network for SH against CC was built using the STRING database (http://string-db.org), and the PPI network was retrieved and subjected to Cytoscape 3.9.1 software to construct the network and obtained hub genes. The functional annotation of GO and KEGG enrichment analyses was carried out by using ShinyGO database (http://www.bioinformatics.sdstate.edu/go/).

#### Compound-Target-Pathway (CTP) network construction

The CTP network was constructed using Cytoscape 3.9.1. software and the concepts of compounds, cross genes, and pathways were introduced respectively.

#### Molecular docking

The phytochemicals compounds of SH were docked with the potential target gene using AutoDock Vina software and Discovery Studio Visualizer was used for visualization of the results. The SDF files of the compounds were obtained from the PubChem database and then converted into PDB file format using OPEN BABEL software. An interactive molecular graphics program called Autodock Vina 1.1.2 was used to perform protein-ligand docking. It calculates and displays the possible docking modes of protein and ligand combinations that are arranged in a hierarchy according to their binding affinities. An active site surrounded a 32 A3 docking array with a 0.375A grid spacing. The free energy binding theory supplied by AutoDock Vina (in kcal/mol) was used to calculate affinity scores, which were then assessed (a higher negative number indicated a stronger binding affinity). A graphical examination was done on the resultant structures and binding docking postures using the DS Visualizer 2.5 or PyMOL Molecular Graphics Framework 2.0 programs to verify the relationships.

#### MD simulation

The compounds with the top five highest binding affinities and the best docking score with AKT1 were then carried on to the MD analysis using the GROMACS 4.2 program [23]. The topology of the ligands file was provided by the SWISSPARAM online server (www.swissparam.ch/), and the protein topological files were constructed using the GROMACS framework. The CHARMM-27 all-atom force field is used to create both topological files [24]. To neutralize the system throughout the simulation, four Na^+^ ions were introduced to the solvated solution. The bond lengths were constrained using the linear constraint solver (LINCS) methodology [25], while the long-range electrostatic interactions were calculated using the particle mesh Ewald (PME) method [26]. The system conducted a 100ps NVT ensemble at 300K for a fixed number of particles, volume, and temperature, following a 100ps NPT equilibration run at 300K and 1 bar of pressure. The temperature and time were set to 300K and 0.1ps for the temperature coupling equilibration [27]. The pressure at 1 bar was calculated using the Parrinello-Rahman barostat algorithm with a time constant of 1 ps [28]. Finally, identical settings were used for 30 ns MD simulations at 300K and 1 bar temperature and pressure, respectively. The root mean square deviation (RMSD), root mean square fluctuation (RMSF), and radius of gyration (Rg) were studied using the generated output trajectory files after the simulation [29]. Further, determining the solvent-accessible surface area (SASA) made it possible to analyze the results of MD simulations and identify major motions based on their amplitude.

#### Binding free energy calculations

Using the aid of GROMACS trajectories, the Molecular Mechanics/Poisson-Boltzmann Surface Area (MM-PBSA) tool was utilized to calculate the binding free energy of the complex. By comparing various energy components, including van der Waals, electrostatic, and solvation energy, MM-PBSA calculates the change in binding free energy [30]. The standard representation of the binding free energy of the protein with ligand in the solvent is as follows [31].

$${\Delta {\text{G}}}_{{\text{binding}}}= {{\text{G}}}_{{\text{complex}}}-({{\text{G}}}_{{\text{protein}}}+ {{\text{G}}}_{{\text{ligand}}})$$

Where G_complex_ provides the total free energy of the complex, G_protein,_ and G_ligand_ reflect the total free energy of the target and the drug separately. The three types of energy were identified using the solvent-accessible surface area (SASA) approach for non-polar energy, the forces field of the MD simulation for the potential energy, and the implicit solvent model for solving the Poisson-Boltzmann (PB) equation for polar energy [32].

#### Principal component analysis (PCA)

Principal component analysis (PCA) has been utilized to investigate the fundamental dynamics of protein-ligand complexes. Using a covariance matrix as a starting point, PCA is a multivariate statistical technique that lowers the data linearly to identify the most prominent features or motions in complex trajectories. The gromacs packages’ gmx_covar module was used to create and analyze a cartesian coordinate covariance matrix to determine the eigenvectors and eigenvalues. Using gromacs’ gmx_anaeig module, the eigenvectors plot of every protein-ligand complex MD trajectory was examined. Finally, the production and visualization of the plots were done using xmgrace [33].

## Results

### Effect of SHPE on cells viability

**Fig. 1:**
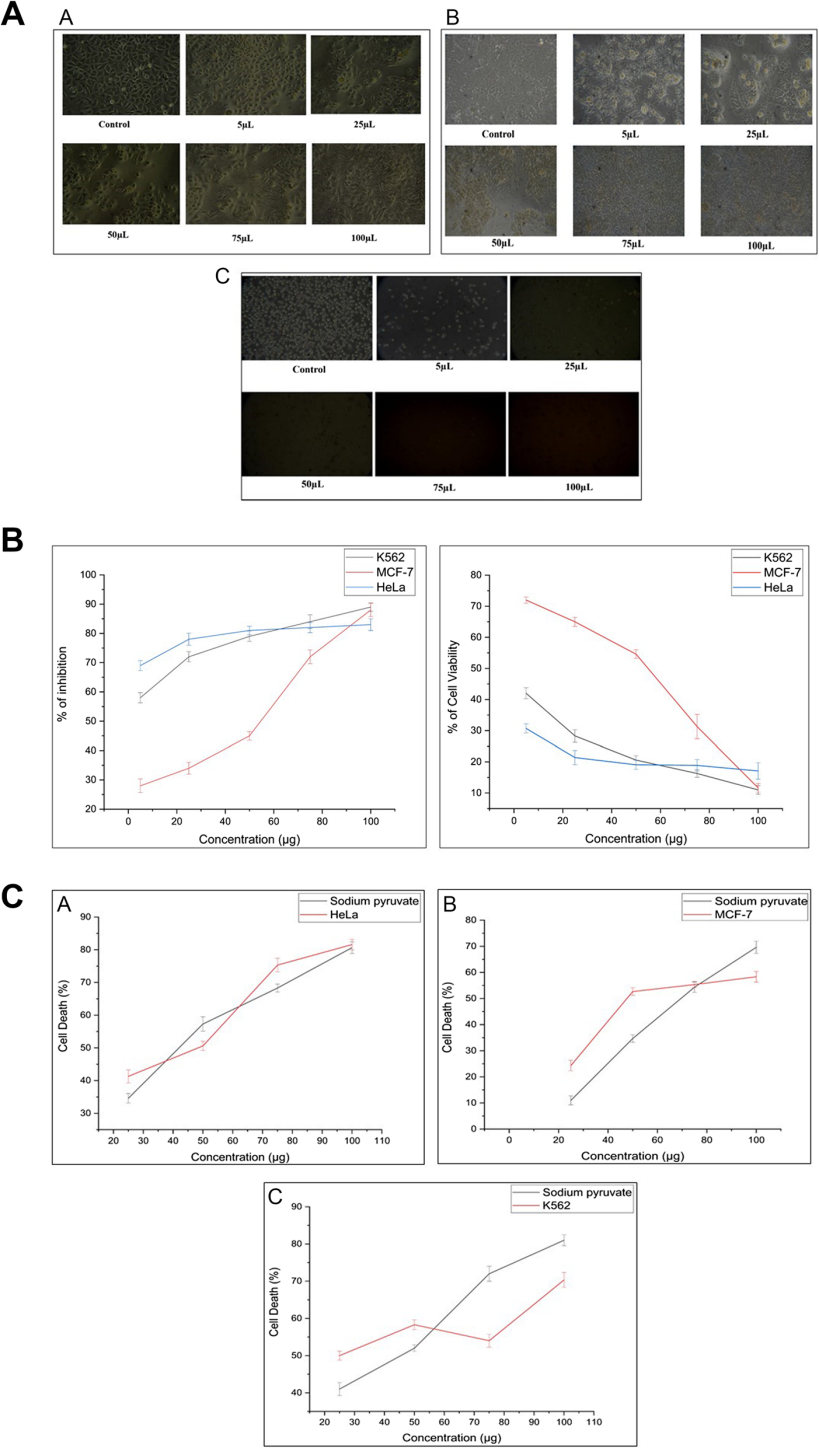
**A** Morphological alterations of cancer cells exposed to plant extract in cell culture medium. A – HeLa; B – MCF-7; C – K-562 cell lines. **B** Percentage inhibition and the percentage cell viability of *S. himalayana tested* against HeLa, MCF-7, and K-562 cancer cell lines. **C** The LDH level of *S. himalayana tested* against A – HeLa; B - MCF-7; and C - K-562 cancer cell lines

The cytotoxic potential of the plant extract can be linked to the presence of various bioactive compounds in the crude extract. In the present study, the cytotoxicity of *S. himalayana* extract at various doses (5, 25, 50, 75, 100 µg/ml) against HeLa, MCF-7, and K-562 cancer cell lines was determined using MTT assay (Fig. 1A). The percentage of cell viability and the percentage of inhibition of the treated cell were plotted against the plant extract at various concentrations (Fig. 1B) and the IC_50_ was calculated. The plant extract showed a significant effect on HeLa, MCF-7, and K-562 cells and it was found that the percentage of growth inhibition increased as the concentration increased. According to the standard evaluation criteria, reactivity is categorized as 0% as none, 1-20% slight, 21-50% mild, 51-70% moderate, and >70% severe. The proportion of inhibition in our investigation was determined to be 69.3, 78.6, 80.9, 81.8, and 82.9%; 27, 34, 45, 71, 88% and 58, 72, 79, 83, and 89% respectively, which were moderate to severe. The percentage of cell viability was concentration-dependent as it showed a decrease in cell viability with an increase in the concentration. Furthermore, the IC_50_ values were found to be 12.01, 50.09, and 20.95 µg/ml. The IC_50_ was found the lowest in the HeLa cell line, followed by K-562, and MCF-7 respectively. Recently, Gordaliza [18] and Salaria et al. [20] showed that the compounds stigmasterol and quercetin had significant anticancer potential against HeLa cells. This explains why HeLa exhibits more inhibition in comparison to K-562 and MCF-7.

The release of lactate dehydrogenase from the cancer HeLa, MCF-7, and K562 cells was measured after the treatment with 0, 25, 50, 75, and 100 µg/ml concentrations of the plant extract and the LDH activity results were shown in Fig. 1C. The IC_50_ values were also calculated and shown in Table 1. The present study showed that the lactate dehydrogenase release was concentration-dependent as the concentration increased, the activity was also increased. The LDH activities were significantly elevated after 48hrs of exposure to the plant when compared to the standard. The results of the LDH assay corroborated the findings of the MTT method.

**Table 1:** The IC_50_ values of plant extract using LDH assays

**Sl No.**	**Species Name**	**LDH IC**_**50**_** (µg/ml)**		
		**HeLa**	**MCF-7**	**K-562**
**1.**	*S. himalayana*	50.25.93±1.15	69.13± 0.79	50.9±1.13
**2.**	Sodium pyruvate	52.04±1.08	65.74±1.21	52.16±1.81

### Evaluation of phytochemical compounds

**Fig. 2:**
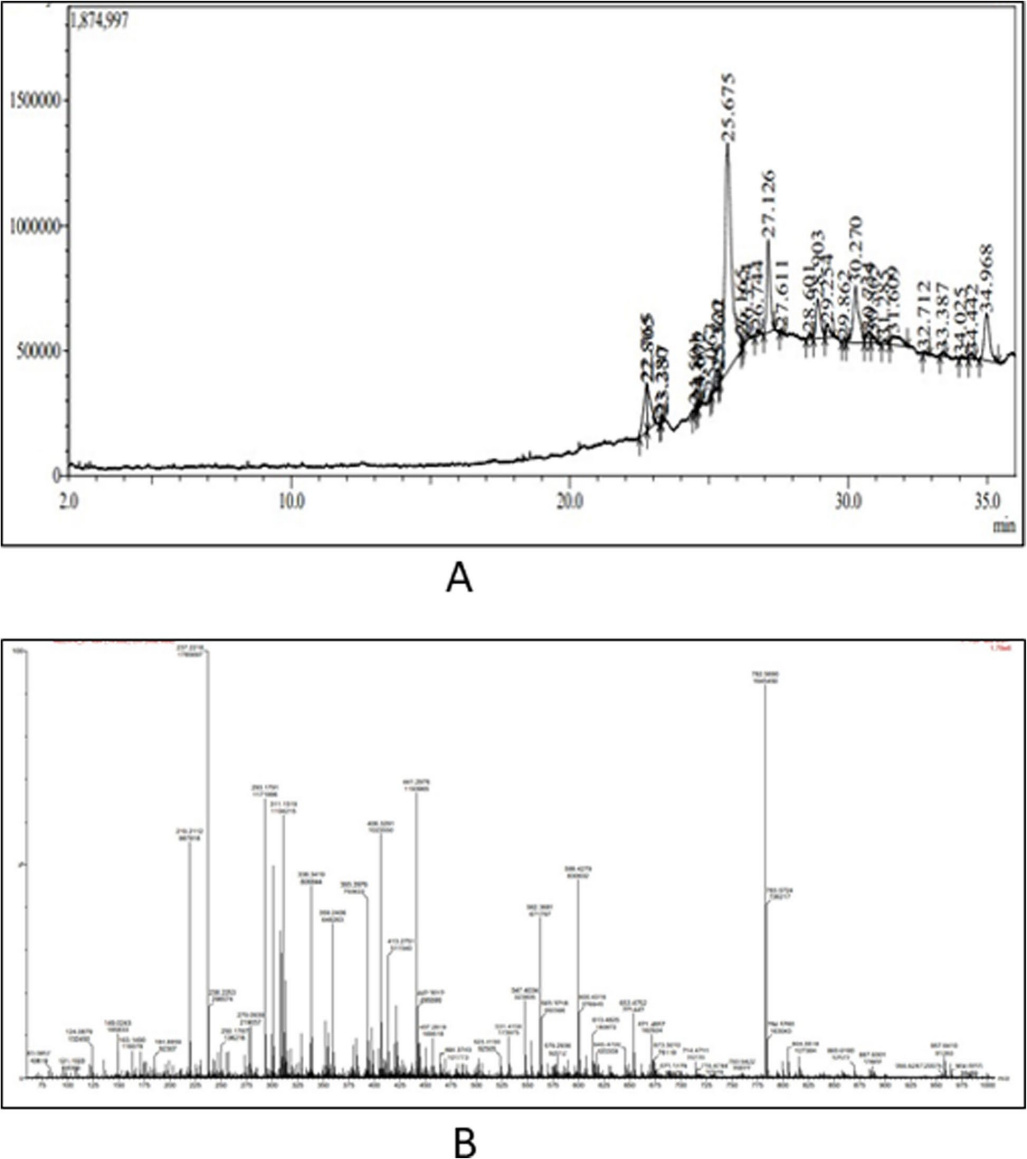
Identification of phytochemicals using GC-MS and LC-MS

Figure 2 displays the presence of various phytochemical compounds in SH. According to the screening of the compounds for their pharmacokinetics properties 14 compounds were identified, including Ergosta-5,7,9(11),22-tetraen-3-ol, (3.beta.,22E)-, Eicosanal, Stigmasterol, Ergoloid mesylate, Quercetin, Lysergamide, Tephcalostan B, Shoyuflavone A, Gentiacaulein, Okanin, Teadenol A, Derriobtusone B, Pongapin, and Mukonal.

### Common targets of SH compounds and CC

**Fig. 3:**
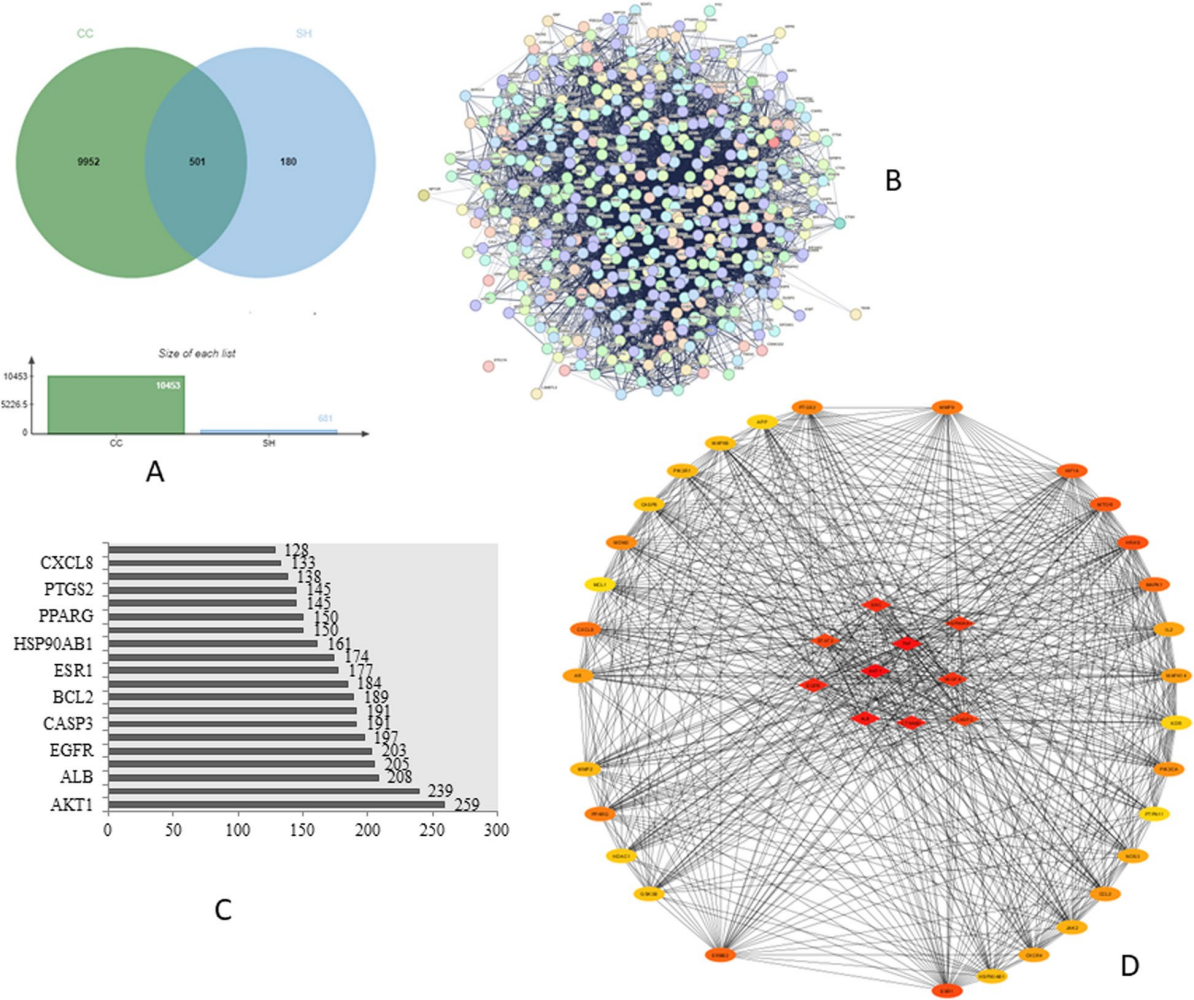
Potential targets of SH compounds against CC and PPI network. **A** Venn diagram of potential targets. **B** PPI network of 501 targets according to the STRING database. **C** The top 10 targets ranked by the degree value. **D** The top 40 potential target networks using Cytoscape 3.9.1

A total of 681 possible SH compound targets were found after examining pertinent targets in the Swiss target prediction database. From the GeneCards database, 10453 targets linked to cervical cancer were searched and compiled. Mutual gene matching between the target genes of the compounds and those of CC resulted in the identification of 501 genes in total, suggesting that the compounds of SH might exert the therapeutic effects of CC via these target genes (Fig. 3A).

### Analysis of PPI network

For the PPI network analysis, a total of 501 projected targets were loaded into STRING (Fig. 3B), 500 nodes and 9929 edges made up the network complex. The network was visualized and examined using the Cytoscape program by determining centrality and other metrics. Following these specifications, all of the targets were organized in circles. The significant role in the network was expressed by the high centrality value, then, the CytoHubba plug-in chose the primary targets (Fig. 3D). The top 10 core target genes were AKT1, TNF, ALB, CTNNB1, EGFR, SRC, VEGFA, HSP90AA1, CASP3, AND STAT3 respectively (Fig. 3C).

### Analysis of GO function and KEGG pathway enrichment

**Fig. 4:**
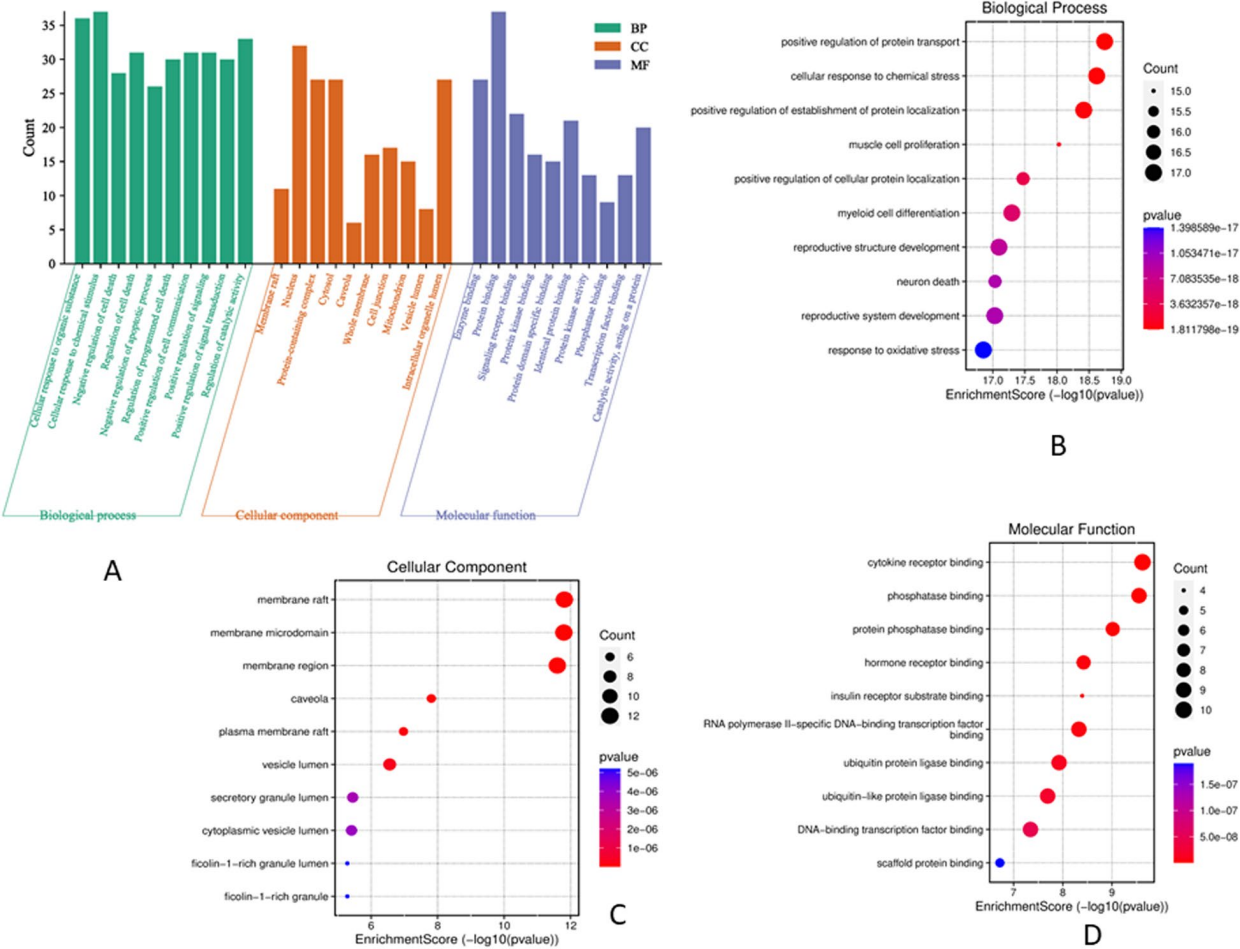
GO function of SH compounds in the treatment of CC. **A** The analysis of GO function, such as biological process (BP), cellular component (CC), and molecular function (MF). **B** Bubble diagram of BP enrichment. **C** Bubble diagram of CC enrichment. **D** Bubble diagram of MF enrichment

**Fig. 5:**
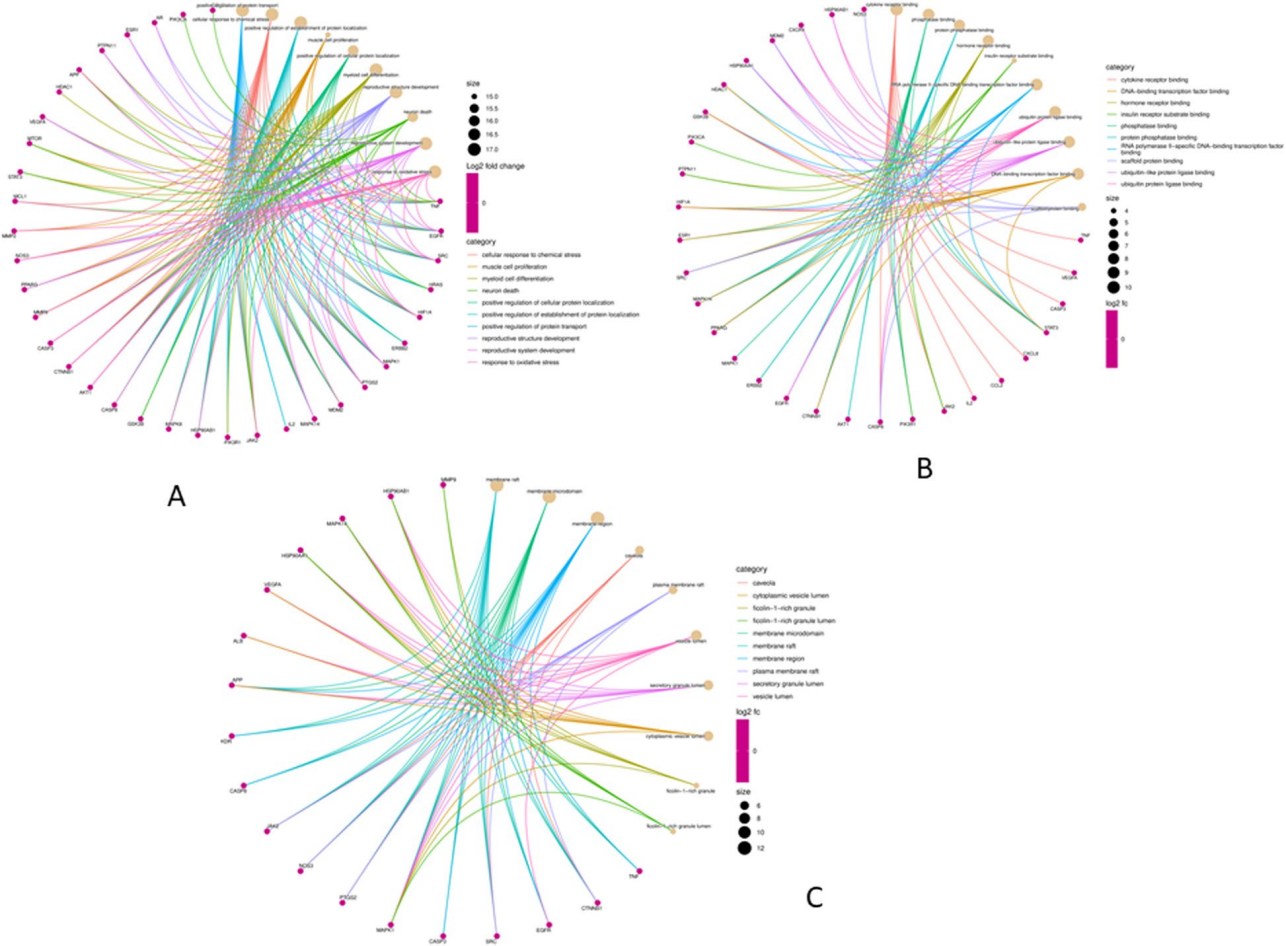
The gene ontology chord of (**A**) BP, (**B**) CC, and (**C**) MF function

**Fig. 6:**
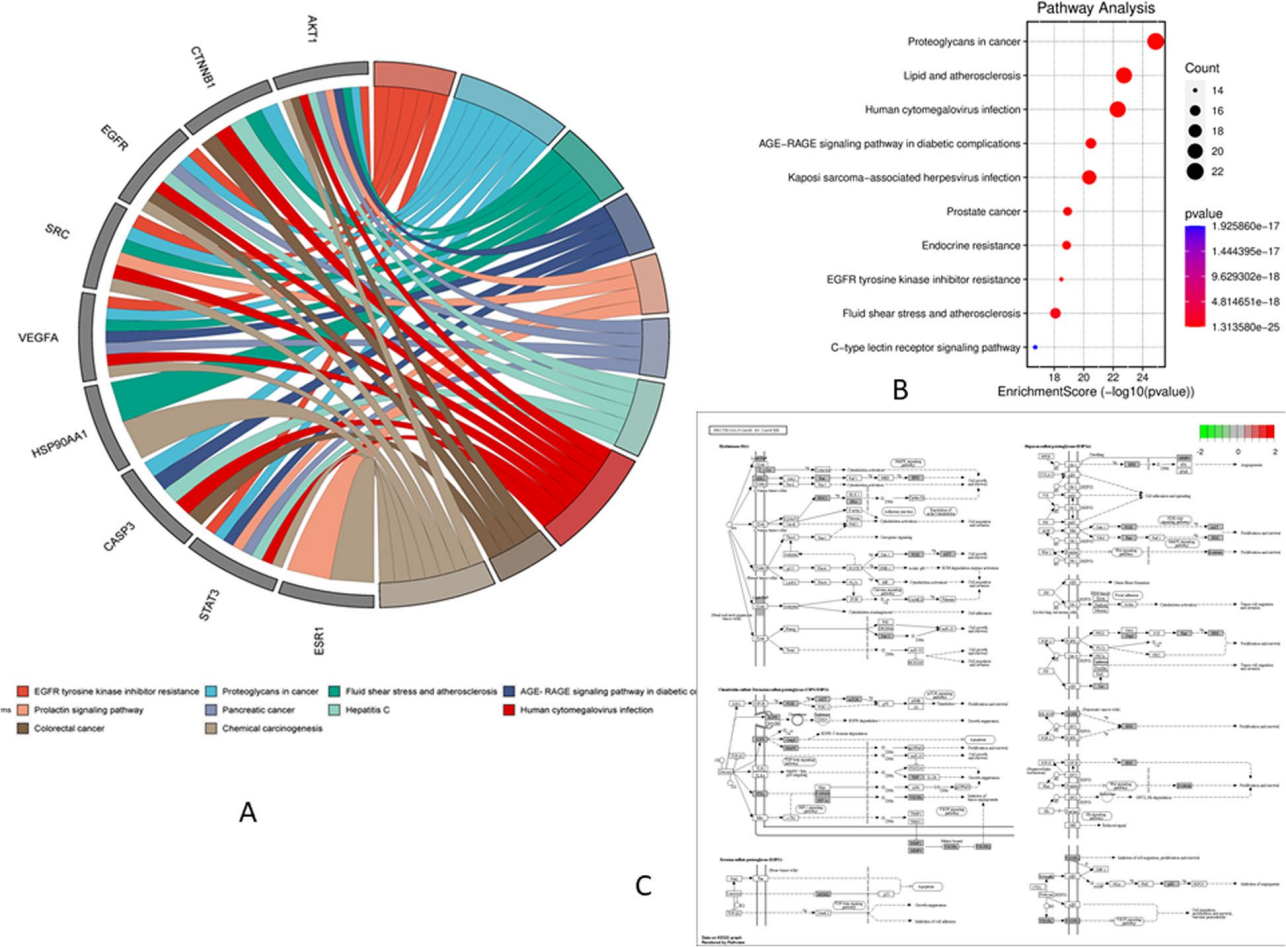
The KEGG pathway enrichment analysis of SH compounds in the treatment of CC. **A** The gene ontology chord of the top 10 pathways of SH compounds against CC. **B** Bubble diagram of KEGG pathway enrichment. **C** The proteoglycan cancer pathway is colored by the KEGG mapper.

A total of 1289 GO items, including 1134 BP (Biological Process), 61 CC (Cellular Component) , and 94 MF (Molecular Function) were acquired from ShinyGo database (*p*<0.01), and the top 10 BP, CC, and MF were chosen for visualization (Fig. 4A). The BP results showed that the activity of active SH compounds in cervical cancer was mostly focused on the regulation of protein transport, cellular response to chemical stress, protein localization, cell proliferation, and differentiation (Fig. 4B). The CC included membrane raft, nucleus, cytosol, cell junction, and mitochondrion (Fig. 4C). The MF mainly included cytokine receptors, phosphatase, hormone receptors, insulin receptor substrate, transcription factor, and ubiquitin protein ligase binding (Fig. 4D). To a certain extent, the various GO functions may also help to explain why the compounds of SH are effective in treating diseases like cervical cancer (Fig. 5). The KEGG pathway enrichment analysis revealed that the compounds were primarily involved in 151 signaling pathways (p< 0.01) and the top 10 enriched pathways are shown in Fig. 6. The proteoglycans in the cancer signaling pathway were the primary pathway of enrichments that included various genes such as MAPK1, MMP2, IL2, MDM2, PIK3CA, STAT3, ERBB2, EGFR, PPARG, PI3K-AKT.

### C-T-P network analysis

**Fig. 7:**
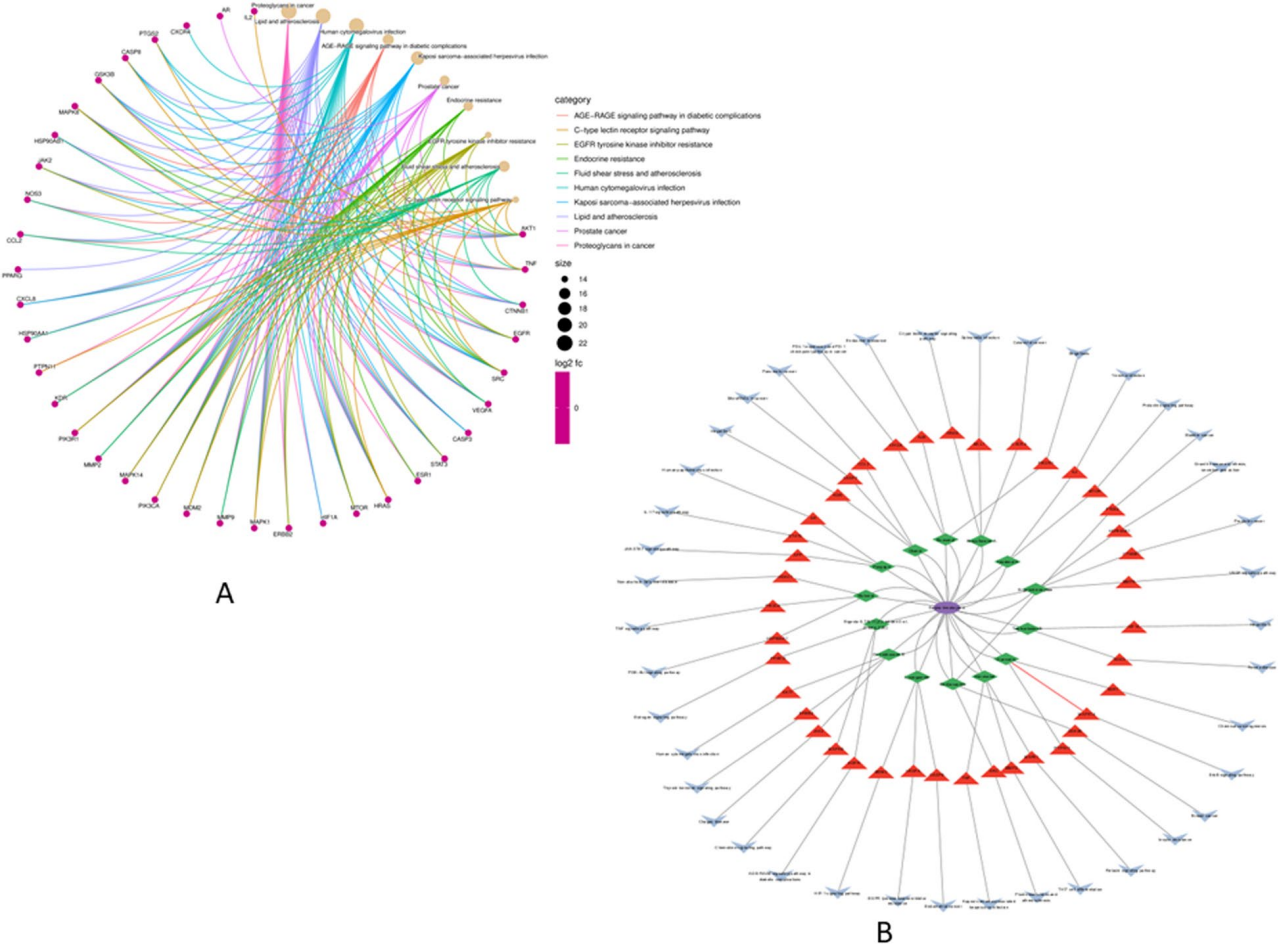
The C-T-P network analysis. **A** C-T-P network construction showed the potential mechanism of SH compounds to treat CC. **B** Cytoscape plug-in was used to construct a C-T-P network

The C-T-P network of the compounds for the treatment of CC is shown in Fig. 7, revealing the complex relationship between the compounds and CC. The plant was shown in purple oval shape, the active compounds were displayed in green diamonds, the target genes were shown in the red triangle, and the pathways were displayed in a blue prism respectively.

### Molecular docking

**Fig. 8:**
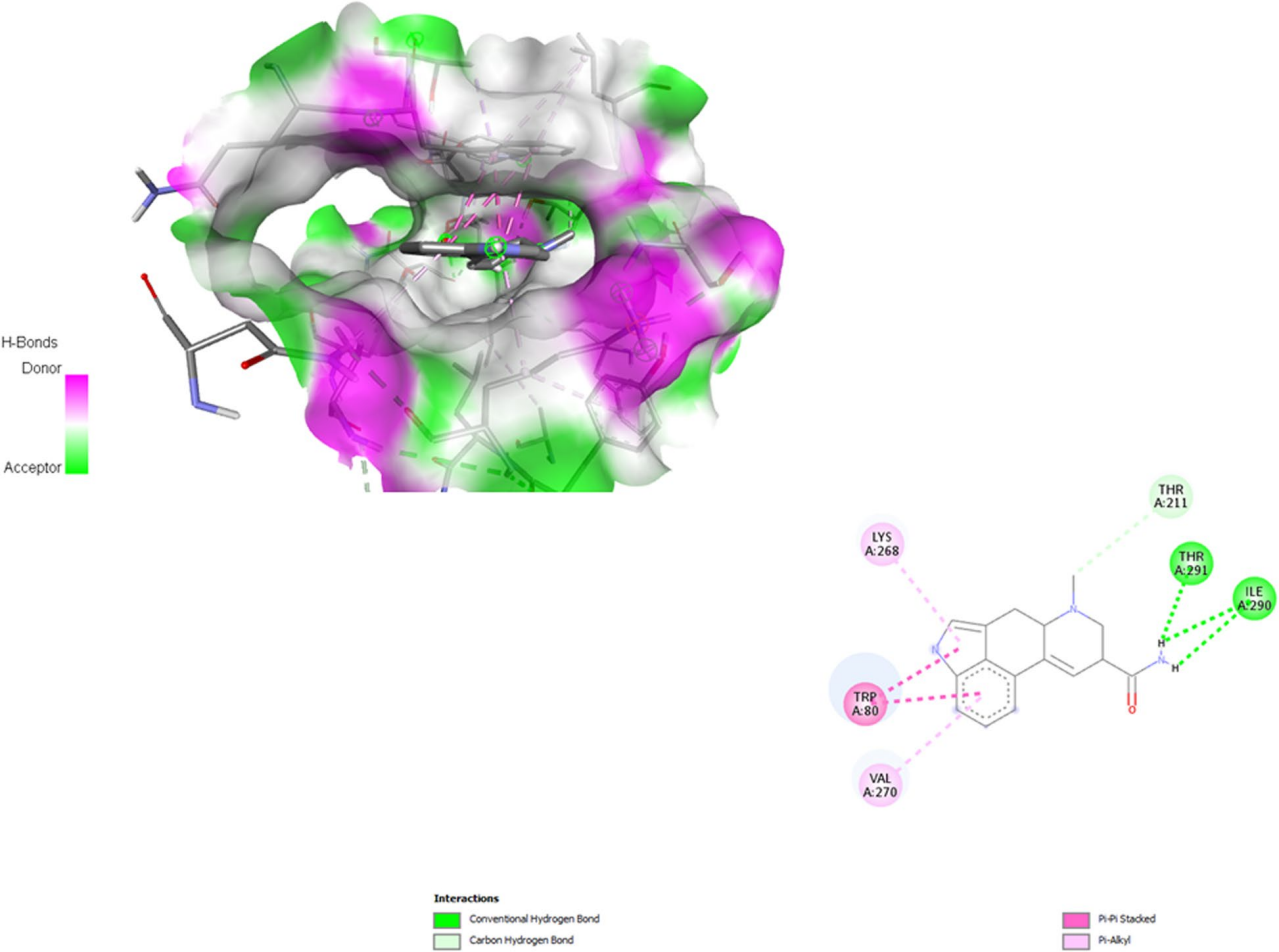
The molecular docking of potential ligands and core target. Lysergamide docking with AKT1

Molecular docking was employed to determine the possibility of binding between the active compounds and the core targets of CC using the AutoDock Vina program (Fig. 8). A previous study demonstrated that a binding affinity of -4.25 kcal/mol signified that the two molecules had a typical level of affinity, whereas -5.0 kcal/mol showed a good binding and -7.0 kcal/mol indicated strong binding affinity [29]. In our investigation, we docked AKT1 with the 14 active compounds of SH. The results showed that Ergosta-5,7,9(11),22-tetraen-3-ol, ergoloid mesylate, and lysergamide had a binding affinity of -15.5 kcal/mol, followed by ergoloid mesylate with the binding affinity of -11.3 kcal/mol respectively (Supplementary Table 1).

### MD simulation

**Fig. 9:**
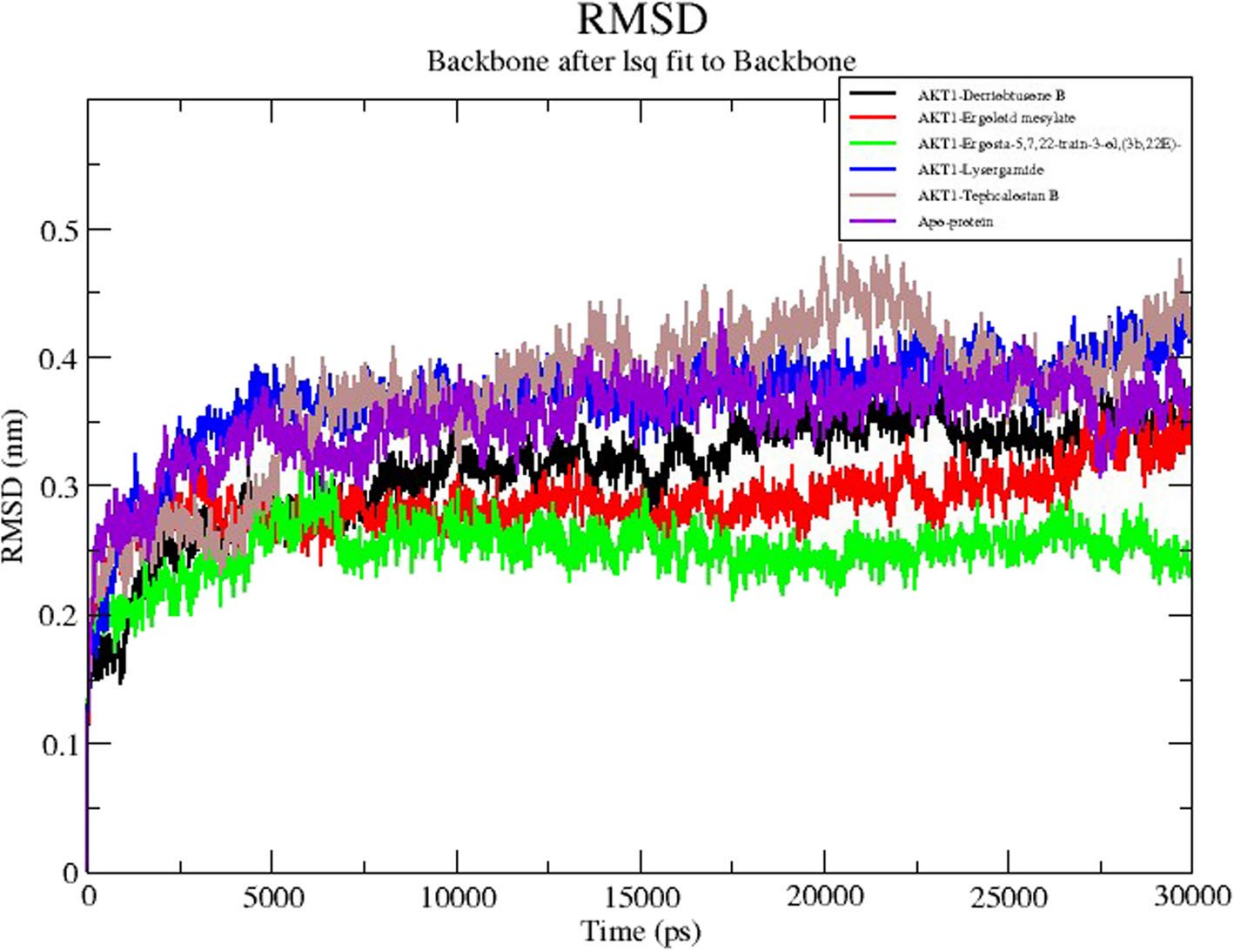
MD simulation of the core target with five potential compounds. RMSD trajectories of five compound complexes with AKT1

The best five protein-ligand complexes in each group such as derriobtusone B (DB), ergoloid mesylate (EM), Ergosta-5,7,9(11),22-tetraen-3-ol (ET), lysergamide (LG), and tephcalostan B (TB) ranked by docking affinity were chosen for MD simulation based on molecular docking results. During the simulation phase, RMSD is a crucial measure to examine the equilibration of MD trajectories and verify the stability of complex systems. To determine whether there was a significant conformational change during the trajectories, the simulations were run within 30ns, and the RMSD of the protein backbone atoms was plotted against time. The root means square deviation (RMSD) of the simulation trajectory was used to determine the stability of the system (Fig. 9). The results showed that the average RMSD values for the complexes of DB-AKT1, EM-AKT1, ET-AKT1, LG-AKT1, and TB-AKT1 were 0.21nm, 0.28nm, 0.25nm, 0.29nm, and 0.27nm respectively, and all the complexes showed almost similar patterns of RMSD changes in the simulations. Stable conformations were attained in all the complexes throughout the period. Overall, all complexes with 5 ligands exhibited RMSD values that were lower than the apoprotein (0.3nm). The temporal plot of the carbon backbone RMSD values shows that all 5 complexes fluctuate by less than 3Å as can be seen in Fig. 9. Despite a significant deviation, the RMSD values for the apoprotein complex remained steady until the end of the simulation. The data suggest that majority of proteins and ligands were stable in their complexes throughout the simulation.

**Fig. 10:**
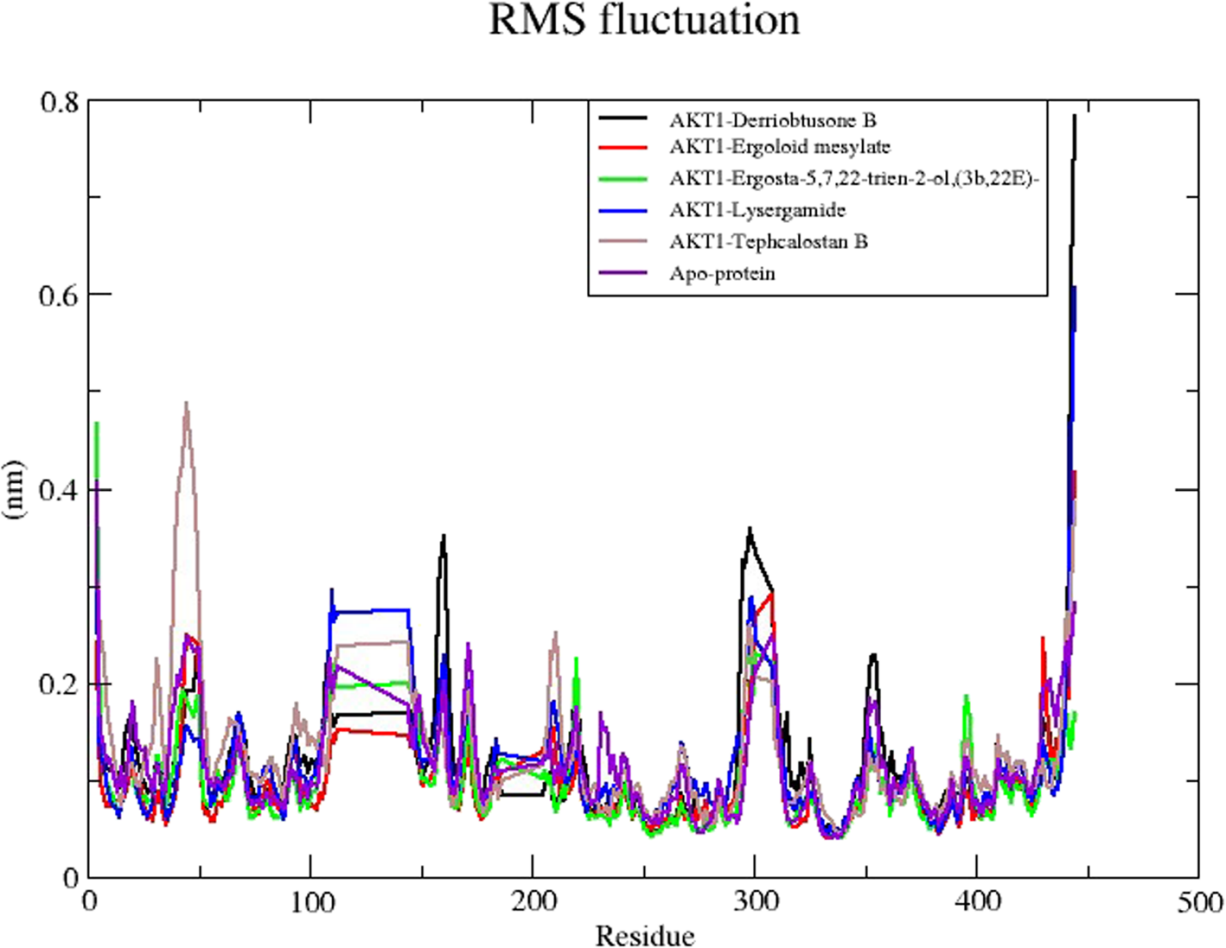
MD simulation of the core target with five potential compounds. RMSF trajectories of five compound complexes with AKT1

The protein flexibility is revealed by the statistical data from RMSF analysis. To represent the fluctuations at the residue level, the RMSF plot was employed. The RMSF profiles of the apoprotein and the complexes calculated by residues index Cα were similar during the simulation. Figure 10 shows that the RMSF values for the complexes revealed higher conformational fluctuations and the highest fluctuation for the residues were found in the EM-AKT1 residue. The average RMSF values of DB-AKT1, EM-AKT1, ET-AKT1, LG-AKT1, and TB-AKT1 were 0.1nm, 0.09nm, 0.08nm, 0.09nm, and 0.087nm respectively. The complexes’ RMSF plot shows a stable binding of the active compounds to the target protein. However, the most stable among the complexes was found in the lowest RMSF value which is the ET-AKT1 complex.

**Fig. 11:**
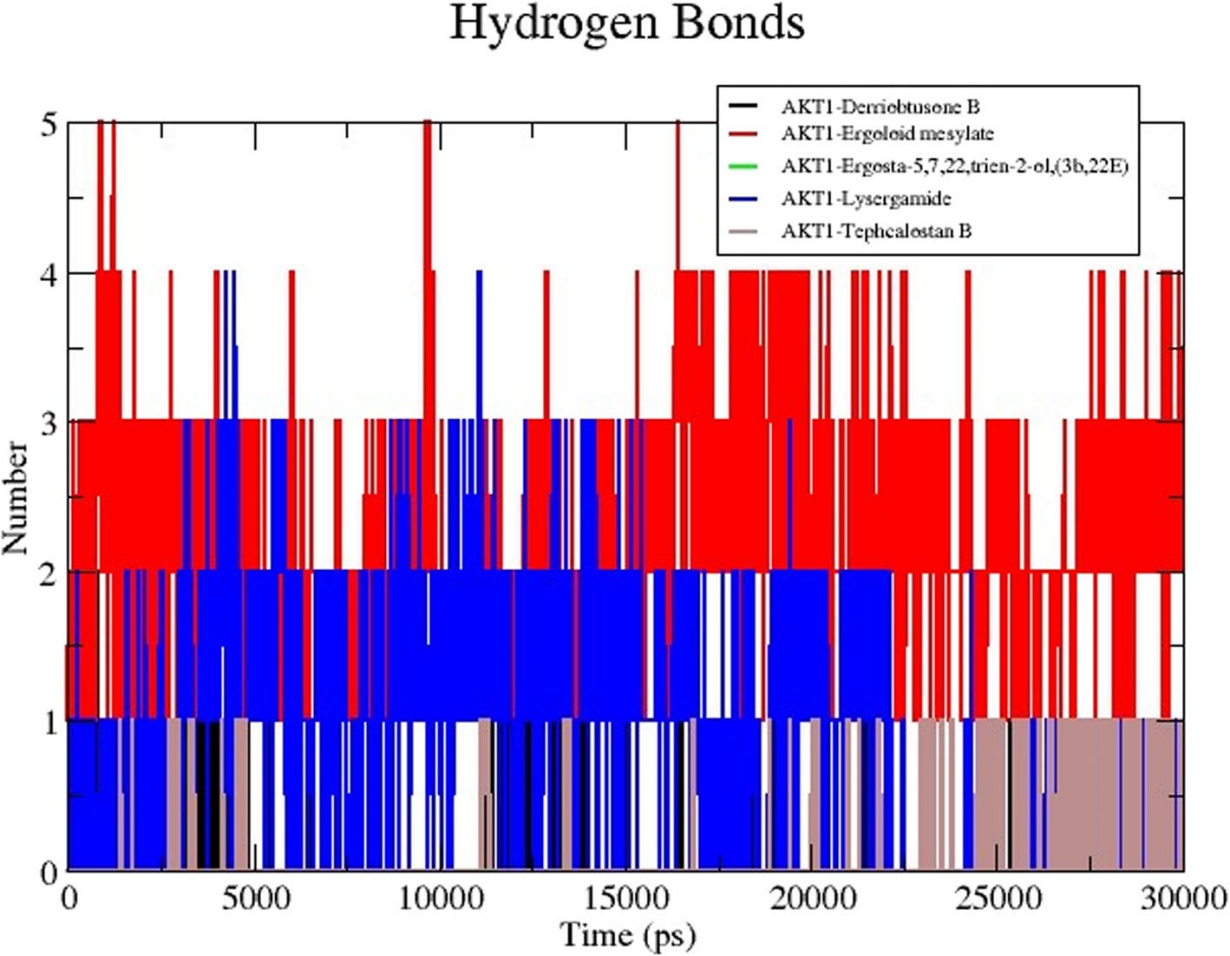
MD simulation of the core target with five potential compounds. Hydrogen bond trajectories of five compound complexes with AKT1

The numbers of H-bonds were calculated during the MD simulations to examine the durability of hydrogen bonds between protein-ligand complexes (Fig. 11). Because hydrogen bonds have a significant impact on the specificity, absorption, and metabolization of drugs, it is generally known that this feature influences medication design. To understand the binding strength of the ligand-protein complexes, we calculated the number of hydrogen bonds generated during the simulation. The average intermolecular hydrogen bonds for DB-AKT1, EM-AKT1, EG-AKT1, LG-AKT1, and TB-AKT1 are 8, 11, 2, 9, and 10, respectively. Among the five complexes, the highest number of intermolecular hydrogen bonds was 11 when EM made a complex with AKT1 protein due to the presence of more carbonyl oxygen atoms. The EM-AKT1 complex, however, generated the fewest intermolecular hydrogen bonds (ET) due to the presence of furan group, which had less van der Waals interaction. During the simulation, all the candidate complexes showed higher hydrogen bond numbers, which revealed their higher stability.

**Fig. 12:**
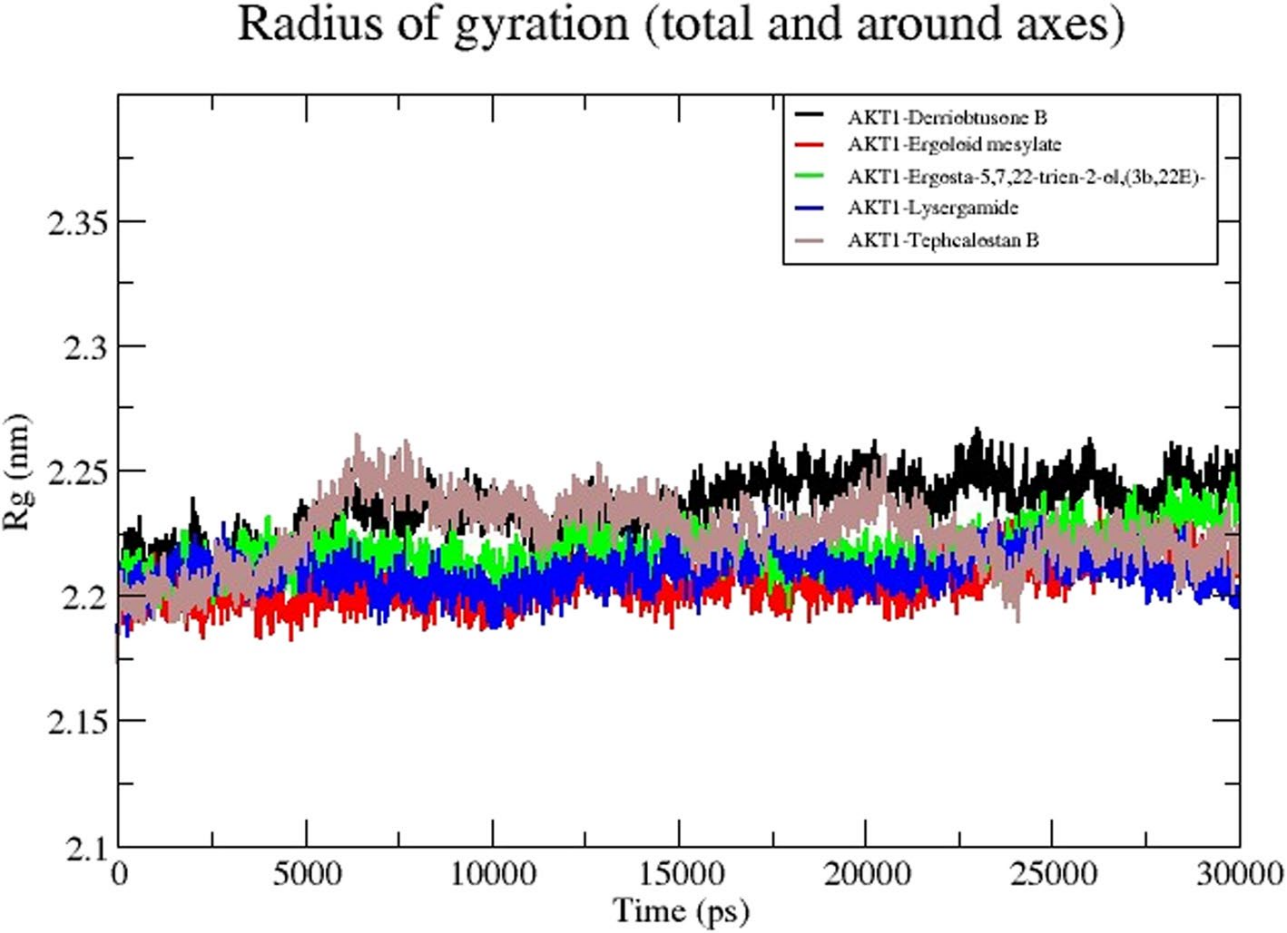
MD simulation of the core target with five potential compounds. Radius of gyration (Rg) trajectories of five compound complexes with AKT1

The radius of gyration (Rg) is a measure of the compactness of a structure. Less fluctuation and consistency throughout the simulation indicate that the system is more compact and stiff. To determine how compact each of the complexes was, we analyzed the Rg values for each of the following complexes such as DB-AKT1, EM-AKT1, EG-AKT1, LG-AKT1, and TB-AKT1 and the average Rg value of each complex were 1.78nm, 1.63nm, 1.74nm, 1.73nm, and 1.85nm respectively (Fig. 12). The highest Rg value was found to be 1.85 for TB-AKT1, while the lowest Rg value was observed in the EM-AKT1 complex. All the complexes had shown consistent fluctuation, which revealed more compactness and higher rigidity.

**Fig. 13:**
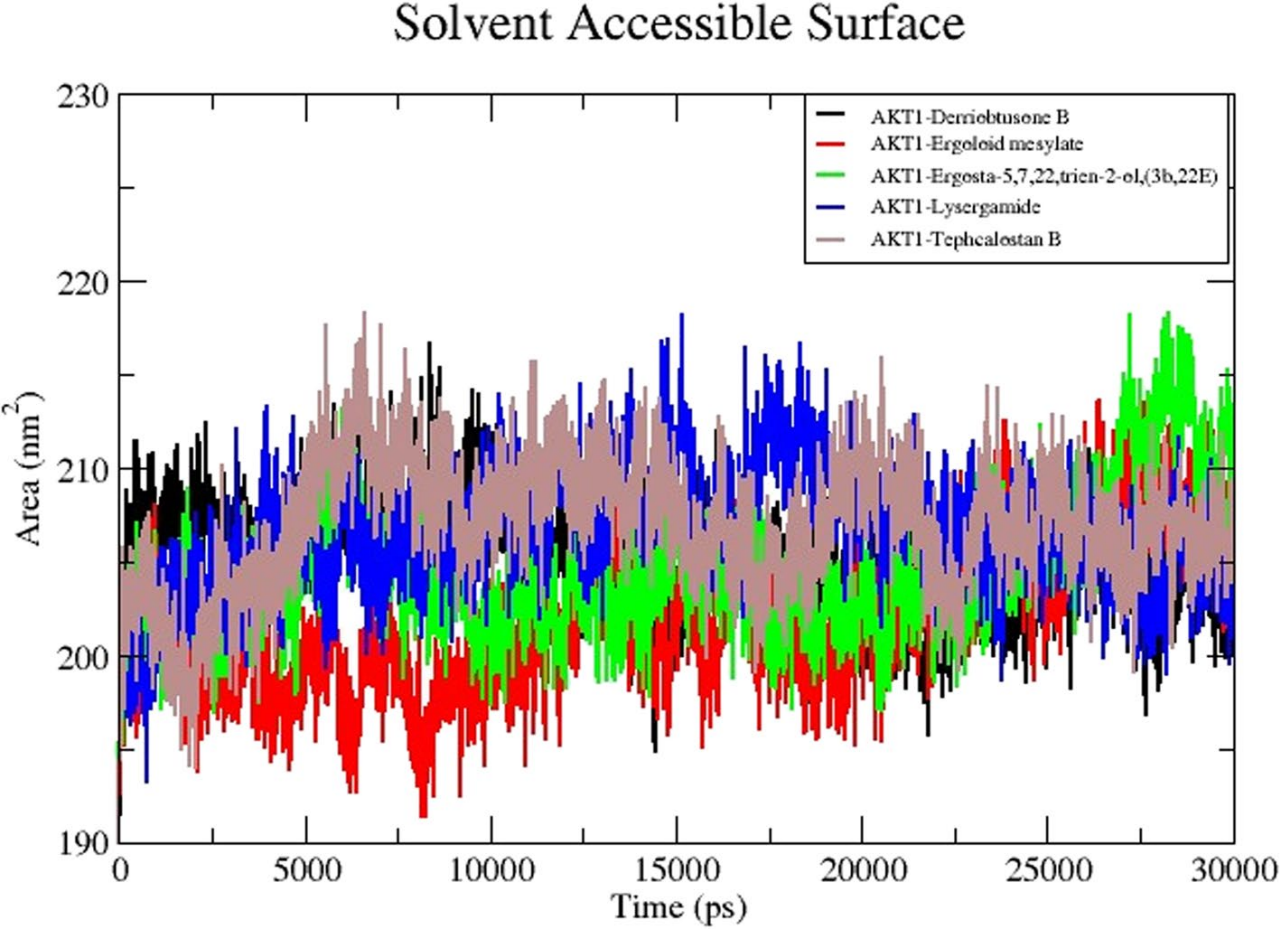
MD simulation of the core target with five potential compounds. Solvent Accessible Surface Area (SASA) trajectories of five compound complexes with AKT1

The amount of protein volume enlarged in each system was then calculated using solvent accessible surface area (SASA) (Fig. 13). The higher SASA values indicate that the protein volume is increased in size with little variation expected across the simulation time. The ligand could change SASA and occasionally have a considerable effect on the protein structure. It was found that the DB-AKT1, EM-AKT1, EG-AKT1, LG-AKT1, and TB-AKT1 had average SASA values of 204.61 nm^2^, 196.30 nm^2^, 202.67 nm^2^, 201.46 nm^2^ and 206.05 nm^2^ respectively. It was inferred from the overall observations that the binding of EM would be able to lessen protein growth.

### Binding free energy calculations

**Fig. 14:**
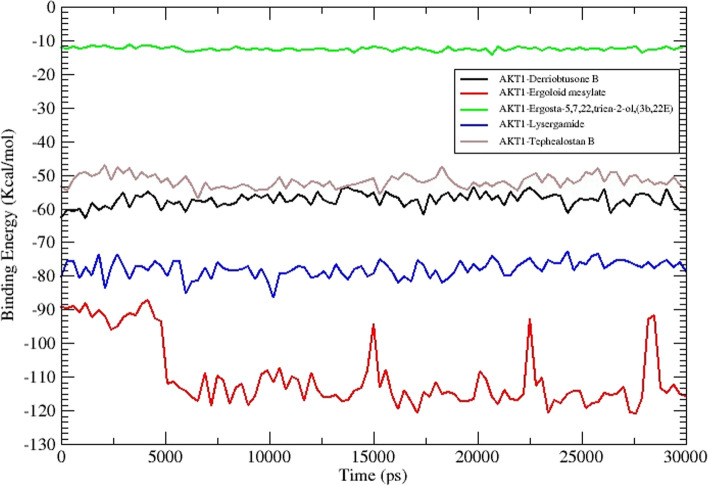
Binding free energy calculation using MM-PBSA

By utilizing MM-PBSA, binding free energy was calculated to gain a deeper comprehension of the stability and molecular interactions of specific therapeutic protein-ligand complexes. Higher stability and a more advantageous energy state during complex formation are indicated by binding free energy that is more negative. MM-PBSA calculates among other forms of energy, van der Waal interactions, electrostatic interactions, and polar and non-polar solvation energy. The MM-GBSA method requires both van der Waals and electrostatic energy components as significant contributors to the calculated binding affinity. Non-covalent interactions between the ligand and receptor are captured, thereby offering valuable insights into the molecular forces that govern the process of binding [34]. By taking into account these energy components in conjunction with solvation and entropic effects, the MM-GBSA method provides a holistic framework for approximating binding free energies and comprehending processes involving molecular recognition [35]. MM-PBSA computations were run at (1ns) intervals on the 30 ns simulation trajectory, using an ionic strength of 0.1M and a solute dielectric constant value of 2. The binding energies were computed using the MMPBSA algorithm to analyze the molecular interactions of DB-AKT1, EM-AKT1, ET-AKT1, LS-AKT1, and TB-AKT1 complexes. The van der Waals, electrostatic, polar solvation, SASA, streptavidin, (SAV, Weeks-Chandler-Andersen (WCA), and binding energies of all complexes were calculated (Table 2). To estimate the degree of conformational change during the interaction, the SASA of the complexes was computed. The interaction between all the complexes was strong, and the MMPBSA binding energy calculations showed high binding energies, particularly in the DB-AKT1 complex, and has been suggested as one of the best active compounds depending on their energy calculations. The van der Waals and electrostatic energies of the complexes as seen in Table 2 and Fig. 14, are potent enough to maintain the active compounds’ ligand interaction with the AKT1 receptor.

**Table 2:** The Van der Waals, electrostatic, polar solvation, SASA, SAV, WCA, and binding energy of ligand-receptor complexes, kJ/mol, were calculated using the MMPBSA method

Energy (kJ/mol)	DerriobtusoneB-AKT1 complex	Ergoloid mesylate-AKT1 complex	Ergosta-5,7,9(11),22-tetraen-3-ol, (3.beta.,22E)-AKT1 complex	Lysergamide-AKT1 complex-24.724	Tephcalostan B-AKT1 complex
Van der Waal Energy	-150.392 +/- 6.784	-128.228 +/- 13.289	-68.66 7 +/- 94.596	-119.127 +/- 12.506	-112.846 +/- 91.635
Electrostatic energy	-12.277 +/- 0.974	-36.800 +/- 13.247	-1.080 +/- 2.105	-24.724 +/- 10.934	-5.915 +/- 9.674
Polar solvation energy	106.187 +/- 6.334	118.623 +/- 21.834	62.142 +/- 101.595	107.096 +/- 17.048	94.700 +/- 98.776
SASA energy	-15.596 +/- 0.709	-15.370 +/- 0.885	-7.979 +/- 10.944	-15.031 +/- 1.094	-11.803 +/- 9.728
SAV energy	0.000 +/- 0.000	0.000 +/- 0.000	0.000 +/- 0.000	0.000 +/- 0.000	0.000 +/- 0.000
WCA energy	0.00 +/- 0.000	0.000 +/- 0.000	0.000 +/- 0.000	0.000 +/- 0.000	0.000 +/- 0.000
Binding energy	-72.311 +/ -3.156	-61.775 +/- 16.688	-15.584 +/- 104.779	-51.786 +/- 14.470	-35.865 +/- 62.974

### Principal Component Analysis (PCA)

**Fig. 15:**
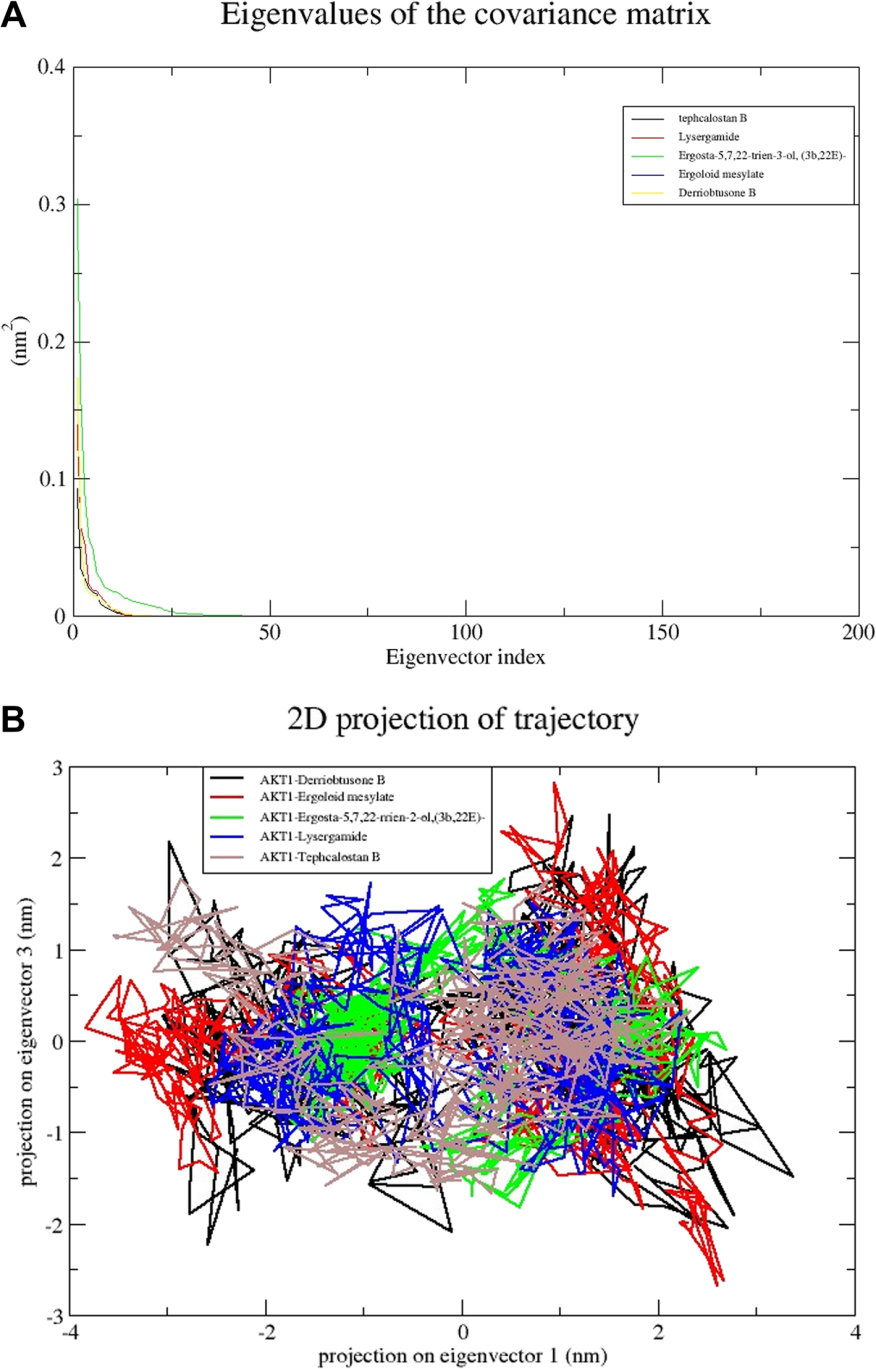
**A** The plot of eigenvalues vs. eigenvector index for DB-AKT1, EM-AKT1, ET-AKT1, LG-AKT1, and DB-AKT1 complexes. **B** The principal component analysis (PCA) of the five compounds in complexes with AKT1 shows PC1 and PC2 for 10 ns simulation trajectories

The PCA analysis was utilized in this study to detect large-scale collective motions in complexes of AKT1 with DB, EM, ET, LG, and TB. The principal component analysis (PCA) or essential dynamics analysis was commonly used to predict the dynamics behaviors of a protein [36]. It is used to forecast the size of big coordinated motion that would occur during ligand binding and it is well known that the initial few eigenvectors characterize the general protein movements. Therefore, we computed the substantially correlated movements for the final 10ns of simulations using the top 50 eigenvectors. After diagonalizing the atomic fluctuations' covariance matrix, eigenvalues were computed. The plot of the eigenvalues for the five complexes against the corresponding eigenvector in descending order is shown in Fig. 15A, and the average values of eigenvalues are 0.03nm^2^, 0.09nm^2^, 0.04nm^2^, 0.06nm^2^, and 0.093nm^2^ respectively. According to the findings, the ET-AKT1 complex is less correlated and more stable than the other four complexes. The first eigenvectors play a significant role in overall movements as shown in Fig. 15B. Therefore, to create a 2D projection chart that more accurately depicts the outcomes, the first three eigenvectors were used. The 2D projection plot reflecting the protein movements in phase space was plotted for the five complexes (Fig. 15B). The plot shows that the ET-AKT1 complex creates a more stable cluster among the studied complexes.

## Discussion

In recent years, both the incidence and mortality rates of CC have grown [37]. Throughout the disease, the biological mechanisms causing the onset and development of CC are constantly changing. The invasion of malignant cancer and metastasis is controlled by a number of genes, which results in a convoluted and polytropic process [38]. To increase patient survival rates and stop the disease from starting in the first place, it is crucial to gain knowledge about the causes and processes of CC development [39].

Plant extracts are naturally rich in bioactive compounds and can induce cytotoxicity that triggers scientists to search for new and innovative therapeutics [40]. A feasible and promising technique for illness prevention and cure is the persistent quest for novel chemicals in medicinal plants and traditional foods [41]. Our study showed that SHPE had significant cytotoxic potential against HeLa, MCF-7, and K562 cells using an MTT assay. According to the United States National Cancer Institute, a crude extract having IC_50_ of 30–40 µg/mL is deemed to have *in vitro* cytotoxic action [42]. The IC_50_ of SHPE extract was found to be 12.01, 46.74, and 20.95 µg/mL respectively, indicating that the extract had cytotoxic capability at even lower concentrations suggesting that it could be a valuable source for anti-cancer medication development. Interestingly, the IC_50_ value is even lower than that of doxorubicin (13 µg/ml), cisplatin (16.68 µg/ml), ifosfamide (993.50 µg/ml) which are currently used drugs for cervical cancer [43, 44]. Due to the complete fragmentation of damaged cells during prolonged incubation with substances, LDH assay is an accurate and reliable marker of cytotoxicity as suggested by various researchers [45]. When compared with the experimental standard, the plant extract-treated HeLa, MCF-7, and K562 cells showed a significantly higher release of LDH. The IC_50_ of carvacrol against HeLa cells and Dichloromethane against human lung cancer have been reported to be 50µg/ml and 58.94 µg/ml [46, 47] respectively, which is comparable to the results observed with our plant crude extract. The inhibition of Hela, MCF-7, and K-562 cell growth strongly proves the cytotoxic nature of SHPE suggesting that this extract could be a potential candidate for new drug formulations for human cervical cancer, breast cancer, and erythroleukemia.

People are beginning to pay more attention to the convergence of computer science and biology as modern bioinformatics emerges. Data mining may assist us in extracting relevant information from large amounts of data and guiding us in conducting meaningful research [48]. NP, which is particularly suited for the complex components and multi-target and multi-mechanism features could be used to study the potential mechanisms of clinical treatment and prevention of diseases based on a large number of experimental data and clinical trial results [49]. In order to gain insight into the mechanism underlying the anticancer potential of the active compounds of SH in CC, this study used network pharmacology to investigate the connections between the drug, targets, and the pathway that leads to those features.

First, we established by the in vitro MTT and LDH assay that the SHPE may effectively suppress the proliferation, migration, and invasion of cancer cell lines. Cell injury or death frequently results in the release of LDH from the cell. The plant extract in this study shows higher levels of LDH activity than the standard, which may indicate that the plant extract causes more cell damage or death. This may suggest that the plant extract is more cytotoxic or efficient against cancer cells than the reference in an anticancer assay. This result could be due to several factors, including the presence of chemicals in the plant extract that are more toxic to cancer cells, increasing cell death, and the subsequent release of LDH. Second, the plant extract may promote cell death by processes (such as necrosis and apoptosis) that are more effective or potent than the standard and increased cell death pathway. It is also plausible that the components in the plant extract are interacting synergistically to enhance cytotoxicity beyond what is noted in the reference standard.

Then, using the Swiss target prediction database, we predicted and screened the active compounds of SHPE targets, using the GeneCards database, we acquired CC targets, and using the Jvenn platform, we obtained common targets of active compounds and CC. Next, we constructed the PPI network using STRING database, visualized it with Cytoscape 3.9.1, and utilized its plug-in CytoHubba to screen and select the target gene called AKT1, for further analysis. The common targets were then subjected to GO and KEGG enrichment analysis to create a pathway-target network. AKT1 was the gene most associated with pathways and MAPK1, MMP2,IL2, MDM2, PIK3CA, STAT3, ERBB2, EGFR, PPARG, PI3K-AKT signal pathways was most associated with targets.

All the compounds that were subjected to molecular docking were safe to use in the future against the AKT1 glycoprotein of human cervical cancer, according to a toxicology investigation that investigated the safety of the possible therapeutic candidate. The results of the molecular docking analysis carried out in this study showed that the majority of the bioactive compounds of SH exhibited a strong binding affinity for AKT1. This was demonstrated by the fact that Ergosta-5,7,9(11),22-tetraen-3-ol, ergoloid mesylate, and lysergamide bound to AKT1 with binding energies of -15.5 kcal/mol, 11.3 kcal/mol respectively. The stability of the ligand-receptor binding configuration and the potential for the interaction increase as the binding energy lowers [50]. These findings imply that AKT1 could be the main target for the bioactive compounds of SH in the treatment of CC. Protein kinase AKT, which is specialized for serine/threonine, plays a crucial role in controlling several cellular processes, including cell proliferation and death. Additionally, it has been demonstrated that the phosphorylation of Akt plays a significant role in HPV-induced cancers such as anal squamous cell carcinomas (ASCC) [51]. According to previous work [52] 67% of ASCCs had p-Akt accumulation inside the cells, and 39 of the 46 cervical neoplasm samples that were examined were positively fixed in formalin and showed p-Akt ibserine 473 [53]. A 48% of cervical cancer patients in stages IB2-II exhibited Akt activation [54]. The SH compound, however, may potentially control other targets to successfully combat CC since they have a significant role in the PPI network and have shown stable binding to the crucial protein’s active pocket.

Using MD simulation is one of the validation techniques for monitoring the signs of the stability of the interaction between a ligand and its receptor. The molecular dynamics investigation of the ligand-protein complex with the best affinity for AKT1 and 5 compounds reveals a stable conformation in water solvation at 310K temperature and 1 atmospheric pressure at 30 ns simulation. The simulation period of 30 ns offers a suitable duration to investigate the range of molecular conformations in the studied system. This period enables the system to collect data from different setups and record significant changes in behavior. A simulation time of 30 ns can produce statistically significant results if the simulation is appropriately equilibrated and sampled [55]. It enables the computation of significant thermodynamic features, such as energy variations, alterations in structure, and interactions between molecules. Nevertheless, prolonging the simulation beyond a given threshold may not inherently enhance the statistical significance of the outcomes, particularly if the system has already achieved equilibrium and the extra simulation duration does not significantly contribute to novel understandings. In general, selecting a simulation time of 30 ns may be seen as ideal for a scientific study as it achieves a balance between computing feasibility, sampling of conformational space, statistical significance, and meeting publishing standards. The metrics that are frequently utilized are RMSD, RMSF, H-bond, Rg, and SASA. The strength and binding affinity of the complexes are influenced by the hydrogen bond. The stronger the bioactivity of the compounds, the more hydrogen bonds are generated during the ligand-receptor interaction. The RMSD values are frequently employed to evaluate the rigidity and stability of the macromolecules [56]. The RMSD values of the complexes remain stable and steadily increase towards the time throughout the simulation. A steady MD simulation result is indicated by an average RMSD value of less than 2Å during the simulation [57]. The flexibility of each amino acid residue and how much it shifts or varies throughout a simulation period are assessed using the RMSF. To examine how the protein-ligand docking complex fluctuates over time, the RMSF was used. The reported lead compound was well bound within the cavity of the target protein binding pocket if the atoms in the active site and the main chain minimally fluctuated, suggesting that the conformational change was limited [58]. The RMSF values of the residues <2Å show that the residue conformation is relatively stable during the simulation [59]. The present findings for all the studied ligands were consistent with the above. The protein-ligand complex’s compactness is measured by the Rg, and its exposure to solvent molecules is determined by the SASA. In turn, each of these characteristics sheds light on the protein-ligand complex’s stability throughout the simulation. The results in Rg and SASA indicate minimal variation, thereby confirming the stability of protein-ligand complexes.

The recommended way to assess a ligand’s stability inside a protein’s binding cavity is through MD simulation, which allows access to a protein-ligand complex’s stability when physiological settings are simulated. The RMSD values of the target proteins remain stable during the simulation. Likewise, RMSF, H-bond, Rg, and SASA also showed a similar pattern, thereby confirming the formation of a stable-ligand complex. The results of this investigation will aid scientists in assessing the substances that are advantageous and effective against human CC.

The present study showed that SH had active compounds such as derriobtusone B, ergoloid mesylate, Ergosta-4,6,8(14),22-tetraen-3-one, lysergamide, tephcalostan B that bind to AKT1 and other genes to prevent the growth of cervical cancer. We included in vitro tests to demonstrate that SH could slow the development of CC by preventing the proliferation and migration of tumor cells. The MTT and LDH experiments proved that SH could inhibit the proliferation of CC.

However, there are a few drawbacks to this study. One drawback was that the amount of compounds in the system pharmacology analysis was not taken into account. A drug’s dosage is very crucial for all kinds of research, including in vitro studies and clinical trials as dosage can affect both the biological mechanism and the cell viability. The lack of consideration for dosages of compounds in medicinal plants has hindered system pharmacology investigations. Research on dose-based in silico herbal medicine will benefit from the finding in this work that compounds with the same scaffold have the same mechanism. Future research will need to provide a methodical pharmacological analysis technique that can quantitatively confirm the amount of compounds. It is crucial to note that findings about cytotoxicity in vitro do not always translate into toxicity in vivo. This could be explained by factors related to pharmacodynamics, pharmacokinetics, and anatomics in both living animals and cell cultures. Further, in vivo toxicity evaluation of the extract is therefore required. It will be interesting in the future to isolate the potential phytoconstituents and test their individual biological and cytotoxic effects against potential target genes and signaling pathways. Also various software might yield varying results since different algorithms are used. However, the disparate results could also be attributed to the use of distinct PDB structures. To address these restrictions, it is proposed that alternative PDB structures be used to represent distinct experimental models and that different tools be used to compare the results. By addressing several targets at once, natural compounds can effectively cure comorbidities and complex disorders. Additionally, another drawback, though, is that because it is challenging to predict the mechanism of natural compounds, it is challenging to apply in the development of novel medications. The findings of the present study are nevertheless novel because it uses network pharmacology and molecular docking to suggest a new natural product mechanism analysis and uses MD simulation to validate secondary drug response.

## Conclusions

The AKT1 glycoprotein is involved in causing human cervical cancer. With the help of bioinformatics techniques such as network pharmacology, molecular docking, and MD simulation, this research project aims to identify the natural compounds of SH that can function as an inhibitor against human CC’s AKT macromolecules in a time and cost-efficient manner. The active compounds of SH were screened using pharmacophore modeling, virtual screening, and molecular docking. The SH contains a variety of phytochemicals, including ergoloid mesylate, lysergamide, quercetin, etc. to prevent human CC by stabilizing the structure and energy of the AKT1 receptor, as demonstrated by MD simulation. Future in vitro and in vivo investigations must validate the chosen compounds. It has been determined that the compounds found in the SH plant are effective against CC.

### Supplementary Information

**Supplementary Material 1.** 

## Data Availability

No datasets were generated or analysed during the current study.
