# Supplementary material for: Identification of novel AKT1 inhibitors from Sapria himalayana bioactive compounds using structure-based virtual screening and molecular dynamics simulations
Source: BMC Complement Med Ther. 2024 Mar 7;24:116. doi: 10.1186/s12906-024-04415-3 (PMC10921764; doi:10.1186/s12906-024-04415-3)
Supplement: Supplementary file 1 — Supplementary Material 1. [file 12906_2024_4415_MOESM1_ESM.docx]

| Compounds | Molecular weight | Rotatable bonds | H-bond acceptor | H-bond donor atoms | Molecular refractivity | Polar surface area | Lipophilicity | Water solubility | Gastrointestinal absorption | Blood brain barrier permeation | P-glycoprotein substrate | Cyt P450 1A2 inhibitor | Cyt P450 2D6 inhibitor | Cyt P450 3A4 inhibitor | Druglikeness (Lipinski rule) | Docking Score (AKT1) (kcal/mol) |
| --- | --- | --- | --- | --- | --- | --- | --- | --- | --- | --- | --- | --- | --- | --- | --- | --- |
| Derriobtusone B | 336.29 g/mol | 3 | 6 | 0 | 88.42 | 71.04Å^2^ | 3.40 | Moderately Soluble | High | Yes | No | Yes | Yes | Yes | Yes | -10.3 |
| Docosanal | 324.58 g/mol | 20 | 1 | 0 | 108.07 | 17.07Å^2^ | 7.63 | Poorly Soluble | Low | No | No | Yes | No | No | Yes | -6.3 |
| Eicosanal | 296.53 g/mol | 18 | 1 | 0 | 98.45 | 17.07Å^2^ | 6.89 | Poorly Soluble | Low | No | No | Yes | No | No | Yes | -6.7 |
| Ergoloid mesylate | 451.71 g/mol | 6 | 6 | 3 | 174.12 | 118.21Å^2^ | 2.59 | Moderately Soluble | High | No | Yes | No | Yes | Yes | Yes | -15.5 |
| Ergosta-4,6,8(14),22-tetraen-3-one | 392.62 g/mol | 4 | 1 | 0 | 126.04 | 17.07Å^2^ | 6.35 | Moderately Soluble | Low | No | No | No | No | No | Yes | -11.3 |
| Gentiacaulein | 288.25 g/mol | 2 | 6 | 2 | 77.02 | 89.13Å^2^ | 2.09 | Soluble | High | No | No | Yes | Yes | Yes | Yes | -8.6 |
| Lysergamide | 267.33 g/mol | 1 | 2 | 2 | 82.88 | 62.12Å^2^ | 1.52 | Soluble | High | Yes | No | Yes | Yes | No | Yes | -10.2 |
| Mukonal | 211 22g/mol | 1 | 2 | 2 | 63.22 | 53.09Å^2^ | 2.36 | Soluble | High | Yes | No | Yes | No | No | Yes | -8.5 |
| Okanin | 288.25 g/mol | 3 | 6 | 5 | 76.36 | 118.22Å^2^ | 1.69 | Soluble | High | No | No | Yes | No | Yes | Yes | -9.4 |
| Pongapin | 336.29 g/mol | 2 | 6 | 0 | 90.25 | 71.04Å^2^ | 3.25 | Moderately Soluble | High | Yes | No | No | Yes | Yes | Yes | -10.8 |
| Quercetin | 302.24 g/mol | 1 | 7 | 5 | 78.03 | 131.36Å^2^ | 1.23 | Soluble | High | No | No | Yes | Yes | Yes | Yes | -9.4 |
| Shoyuflavone A | 386.31 g/mol | 6 | 9 | 4 | 95.56 | 154.5Å^2^ | 1.04 | Soluble | Low | No | No | No | No | No | Yes | -9.6 |
| Teadenol A | 276.24 g/mol | 1 | 6 | 3 | 68.47 | 96.22Å^2^ | 1.22 | Soluble | High | No | No | No | No | Yes | Yes | -8.3 |
| Tephcalostan B | 348.31 g/mol | 1 | 6 | 0 | 94.31 | 71.04Å^2^ | 3.76 | Moderately Soluble | High | Yes | No | Yes | Yes | No | Yes | -11.1 |

**Supplementary Table 1**. The binding affinities, molecular properties, physicochemical and pharmacokinetics of the bioactive compounds
